# Nanorobots to Treat *Candida albicans* Infection

**DOI:** 10.34133/research.0455

**Published:** 2024-08-15

**Authors:** Yanling Hu, Guisheng Zeng, Yue Wang, Dongliang Yang

**Affiliations:** ^1^ College of Life and Health, Nanjing Polytechnic Institute, Nanjing 210048, China.; ^2^ A*STAR Infectious Diseases Labs (A*STAR ID Labs), Agency for Science, Technology and Research (A*STAR), 8A Biomedical Grove, #05-13 Immunos, Singapore 138648, Singapore.; ^3^ Department of Biochemistry, Yong Loo Lin School of Medicine, National University of Singapore, Singapore 117597.; ^4^ Key Laboratory of Flexible Electronics (KLOFE) and Institute of Advanced Materials (IAM), School of Physical and Mathematical Sciences, Nanjing Tech University (NanjingTech), Nanjing 211816, China.

## Abstract

*Candida albicans* is an opportunistic fungal pathogen of humans. It causes a variety of infections ranging from superficial mucocutaneous conditions to severe systemic diseases that result in substantial morbidity and mortality. This pathogen frequently forms biofilms resistant to antifungal drugs and the host immune system, leading to treatment failures. Recent research has demonstrated the potential of nanorobots to penetrate biological barriers and disrupt fungal biofilms. In this perspective paper, we provide a brief overview of recent breakthroughs in nanorobots for candidiasis treatment and discuss current challenges and prospects.

Annually, 65 million invasive fungal infections occur worldwide, resulting in approximately 3.8 million deaths [1–3]. Among these fatalities, about 80% is attributed to fungal sepsis from hospital-acquired fungal infections, with *Candida albicans* being the most prevalent culprit [2,4]. Current treatments for invasive candidiasis mainly involve 3 classes of antifungal drugs, which target different components of fungal cells [5]. For example, polyenes bind to ergosterol on the fungal cell membrane and induce membrane perforation; azoles block ergosterol biosynthesis; and echinocandins inhibit the synthesis of β-glucan, a component of the fungal cell wall, by targeting 1,3-β-glucan synthase [2]. However, the widespread occurrence of candidiasis and the excessive use of antifungal drugs have led to the rise of drug-resistant *C. albicans*. Furthermore, *C. albicans* can develop intricately structured biofilms under specific conditions, bolstering their resistance against both antifungal agents and the host’s immune defenses. This resistance leads to frequent treatment failures and elevated mortality rates [6,7]. Therefore, there is an urgent need to devise innovative therapeutic strategies to combat *C. albicans* infections.

Nanorobots are nontraditional nanoparticles. They are minuscule machines that can move autonomously using internal or external-driving forces to perform specific tasks [8,9]. As nanotechnology progresses rapidly, nanorobots are undergoing diverse structural designs to enhance their functionality in physiological settings. Furthermore, these nanorobots can be equipped with augmented permeability, environmental responsiveness, drug delivery capabilities, multimode antifungal collaboration, and robust biosecurity via surface modifications or packaging [10–12]. Currently, these tiny robots have demonstrated promising outcomes in treating fungal infections (Figure) [5,13]. For example, Ji et al. [5] recently developed laser-activated nanorobots to treat subcutaneous *Candida* infections. In this work, poly(divinylbenzene) thermal insulation material was applied to one side of platinum nanoparticles with photothermal conversion properties, creating parachute-like Janus nanorobots (PJNs). The antifungal drug miconazole nitrate (MN) was loaded onto the poly(divinylbenzene) canopy through hydrophobic interactions. In vitro studies have shown that, under light excitation, PJN can increase MN uptake by fungi and improve MN adsorption by fungal biofilms due to the autonomous motion effect of PJN induced by thermal gradients. Even more excitingly, PJN-MN, when assisted by a laser, can penetrate the skin barrier, efficiently delivering MN to the infected dermis. This achieves effective treatment of subcutaneous *Candida* infection through a combined action of the drug and photothermal therapy.

**Figure.**
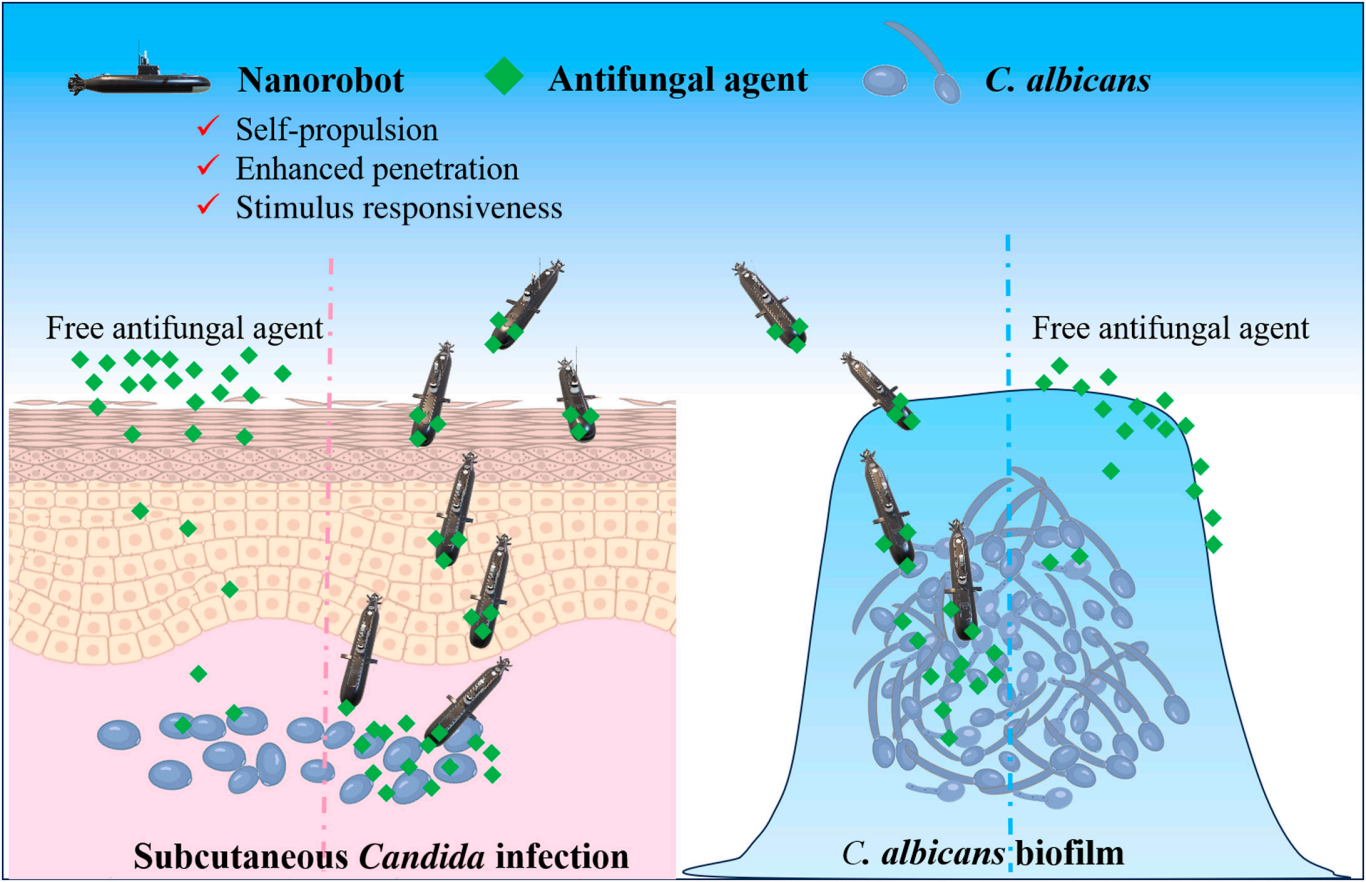
Nanorobots for *Candida* infection treatment.

*C. albicans* can colonize and grow on the surface of medical implants, forming biofilms that lead to infections and potentially cause the implants nonfunctional [14,15]. To combat biofilm formation on implants, clinical interventions such as surgical debridement are commonly used. However, this approach increases medical costs and places heavy physical and psychological burdens on patients. Recently, Oh et al. [13] developed a nanorobot capable of erasing fungal biofilms and targeting the production of reactive oxygen species (ROS) through nanozyme-based catalytic reactions to remove pathogenic fungi. In their design, peroxidase-like iron oxide magnetic nanoparticles (IONPs) catalyze the formation of hydrogen peroxide at the biofilm infection site, generating ROS, such as hydroxyl radical, to eliminate pathogens and their biofilms. The researchers skillfully utilized electromagnetic fields on both *x* and *y* axes to achieve programmable assembly of IONPs, constructing various IONP aggregates, including spherical or sea-urchin shapes. Furthermore, by adjusting the frequency of the *X* and *Y* electromagnetic fields and using spatiotemporal management, the researchers can control the motion behaviors of IONPs, such as rolling, gliding, vibrating, and dabbing, as well as their movement speed. This control also effectively modulates the catalytic activity of IONP aggregates. Dynamic motion can markedly activate IONP aggregates, leading to increased ROS production, with the production rate being directly proportional to their linear velocity. For instance, when IONP aggregates are stationary, their peroxidase-like activity is minimal. However, their catalytic activity increases substantially with motion, being 7 times higher in rolling motion and 6 times higher in sliding motion compared to vibration. By utilizing these different motion modes, IONPs can be precisely delivered to the site of infection, enabling in situ ROS generation and enhanced ROS diffusion. ROS can cause oxidative damage to intracellular bioactive molecules, thereby exerting antifungal activity. Antifungal assays have shown that IONP nanomachines can specifically target *C. albicans* biofilm through a painting and dabbing effect, accumulating on the surface of *C. albicans* to effectively inactivate the fungal biofilm. In addition, *C. albicans* biofilms on explant mucosal tissues can be effectively and accurately cleared using magnetic-field-mediated antifungal therapy with IONPs. Because of the magnetic field’s robust tissue penetration capabilities, magnetic-field-controlled nanorobots are well suited to meet clinical requirements [16].

The studies suggest that nanorobots hold high potential for treating *Candida* infections by enhancing the delivery of antifungal agents to the infection site and can be combined with nondrug therapies to eliminate drug-resistant fungal pathogens. The nanorobot-based delivery technology offers fresh perspectives in overcoming the biofilm matrix, skin, and tumor barrier, especially deep drug delivery. Although in vivo and in vitro assessments have confirmed the considerable promise of nanorobots in the treatment of infectious diseases, there are several issues that should be considered. First of all, most researches on antifungal nanorobots have been conducted in vitro or in mice. Since human fungal infections differ markedly from fungal behaviors and pathogen–host interactions observed in these models, current studies may not fully capture the complex interactions between nanomachines, fungal pathogens, and humans. Second, large-scale production, cost, and biosafety of nanorobots are critical considerations. Although nanorobots have made breakthroughs in the treatment of bacterial infections, their transition to clinical use is still hampered by the absence of large-scale, standardized production processes. To expedite the industrial application of nanorobots, the development of scalable synthesis methods is an urgent priority that must be addressed to unlock their true economic and societal advantages. Last, effective delivery and tracking of nanomachines at the infection site in vivo remain challenging for clinical applications. Because of constraints such as their limited size and inadequate driving force, nanorobots cannot get rid of the fetter of viscous biofluids in fungal biofilms or body tissues, nor can they overcome the barriers presented by body compartments. Consequently, the development of nanorobots with smaller sizes and stronger driving forces represents a key area of future advancement. Moreover, leveraging the photothermal, magnetic, and other photophysical properties inherent to the nanorobots, researchers can use techniques such as photoacoustic imaging and magnetic resonance imaging to monitor the in vivo delivery of these nanotherapeutics [17]. The development of biofilm probes to visualize the bacterial biofilm clearance process plays a crucial role in guiding the treatment of biofilm infections. Throughout the treatment regimen, medical professionals can determine whether to induce other treatment modalities (such as antibiotic treatment) to remove the biofilm debris leftover from nanorobots treatment, according to the actual needs. Addressing these issues could enable nanorobots to greatly enhance antifungal drug penetration and targeting, offering promising solutions for treating fungal infections.
